# Energy Absorption Behavior of Al-SiC-Graphene Composite Foam under a High Strain Rate

**DOI:** 10.3390/ma13030783

**Published:** 2020-02-08

**Authors:** Sourav Das, Dipen Kumar Rajak, Sanjeev Khanna, D. P. Mondal

**Affiliations:** 1 Department of Mechanical and Aerospace Engineering, University of Missouri, Columbia, MO 65211, USA; sourav328@live.com (S.D.); KhannaS@missouri.edu (S.K.); 2 Department of Mining Machinery Engineering, Indian Institute of Technology (ISM), Dhanbad 826004, JH, India; 3 Department of Mechanical Engineering, Sandip Institute of Technology & Research Centre, Nashik 422213, MH, India; 4 CSIR-Advanced Materials and Processes Research Institute, Bhopal 462026, MP, India; mondaldp@yahoo.com

**Keywords:** graphene, metal foam, high strain, energy absorption, plateau stress

## Abstract

The present work was addressed to the closed-cell aluminum (Al)-silicon carbide (SiC) particles (15 wt.%) with graphene (0.5 wt.%) reinforced hybrid composite foam, which was produced through the melt route process. Under the strain rates ranging from 500 s^−1^ to 2760 s^−1^, the compression deformation behavior of hybrid composite foam was executed. The compression results disclosed that plateau stress along with energy absorption of produced hybrid composite foam are heightened with strain rates and is also discovered to be responsive to the relative density under the confront domain of experiments. Analysis of Variance was deployed for optimizing parameters such as strain rates, mass, density, relative density, and pore size. Furthermore, the contribution of each optimized parameters on plateau stress and energy absorption were observed.

## 1. Introduction

Metal foams are turning out to be potential materials for multifunctional or structural applications based on the degree of openness or porosity, and their excellent compounding of physical and mechanical properties including lightweightness has also been disclosed [1]. The inherent cellular structure of metal foam showed exceptional damping ability [2], better sound absorption [3], along with the ability to integrate sudden impact and thrust [4,5]. It was reported that Al-alloyA356/SiC composites depicted better sound absorption properties than Al foam and a higher amount of Al/SiC interface reasoned for larger sound absorption [2]. The deformation characteristics of the foam due to high-velocity impact exhibited a linear relationship with shock waves [5]. Attempts were also executed in structural applications for applying the closed cells Al-foams in a core of sandwich structure [6,7,8,9,10]. On the other hand, open-cells foams were proven to be an excellent and suitable material for applications in heat exchangers [11,12], filters [13,14], etc. Metallic foams when subjected to instantaneous impacts transform impact energy into plastic energy and are further able to absorb more energy as comparable to the corresponding bulk solid materials. Due to these characteristics, it was intended to apply metallic foams in an automobile for the roofing structure, front hood, bumpers, etc. to absorb energy throughout the crash [15]. In recent days, many companies are making metal foam for various applications, namely lightweight structures, biomedical implants, catalysts, and electrodes [16]. The knowledge of metal foam compressive response at higher strain rates is demanded for applying them to absorb impact energy [17,18,19,20,21,22,23]. The closed cell Al-foam features potential application in crash box. Aluminium foam material is tucked inside the crash box in order to increase the ability to absorb energy [24,25,26,27,28,29]. Paul and Ramamurty [30] observed that the deformation of closed-cell aluminium foam at strain rates in the ranges from 10^−1^ s^−1^ to 10^−5^ s^−1^ is enhancing noticeably the capability of the material to absorb energy. In frontal impacts that occurred during the crash events, the crash boxes are required to break down to absorb energy preventing rear body parts and the main cabin frame from the damage and passengers could be spared with minimal injuries. The ability to absorb energy by aluminium foam material can be determined by the area under the curve in a stress–strain diagram; the wider the plateau region, the higher the energy absorption. In addition, it is important to understand low and high strain rate behavior for assessment of enhanced energy absorption and crashworthiness behavior [31,32,33,34,35,36,37]. Myers et al. studied about material which strongly depended on the chemical composition of metal matrix composite (MMC) and also disclosed heat treatment conditions, and explored stiffness and fracture mechanism under quasi-static loading and high strain rate [35]. Luong et al. [38] have not encountered that A4032 matric alloys are more influenced by strain rate sensitivity, and A4032/fly ash cenospheres having measurable strain sensitivity and high degree of energy absorption capacity at high strain rate as compared to a quasi-static strain rate. It was reported that higher mechanical strength obtained in the composite metal foam at high strain rate deformation is mainly attributed to strain rate sensitivity and entrapped gas pressurization [39,40,41,42,43,44]. Orbulov and Nemeth [41] studied carbon fiber reinforced metal matrices and also the comportment of Al_4_C_3_ crystals caused by large scatter in the mechanical properties, whereas ultimate tensile strength having a low impact but compressive strength was remarkable. Moreover, the fracture surfaces of MMCs have investigated the composite disclosed rigid fracture and more bending strength by increasing carbon fiber volume fraction. Peroni et al. [44] investigated the iron matrix syntactic foams for their potentiality in applications consisting of a high degree of energy dissolution in a constrained space. Researchers have collected several amounts of data on material distortion of aluminium foam subjected to compression at elevated strain rates [45]. In another study [46], closed-cell Al-foam was studied under quasi-static (strain rate at 10^−3^ s^−1^) and dynamic strain rates (750 s^−1^) utilizing an SHPB (Split Hopkinson Pressure Bar) apparatus. Their study reported that plateau stress and energy absorption increased along with relative density and strain rates. 

Graphene is an allotrope of carbon configuring single layer of atoms in a 2D lattice structure. It expeditiously mediates flow of heat and electricity, carrying enormous strength compared to steel graphene exhibits flexibility equivalent to rubber [47]. Infusion of graphene nanoplatelets in the metal matrices structure of metal matrix composites (MMCs) improves the material strength. The latest research has shown enhancement in the material strength by 62% when aluminium alloys were reinforced with 0.3% of graphene [48]. The strengthening mechanism of the graphene dispersed composite was reported as due to successful load transfer from the metallic matrix to the graphene sheets through their interface. However, in order to produce MMCs with reinforcement of graphene nano-platelets in the mass quantities, it is tremendously difficult to efficiently integrate and distribute graphene nanoplatelets uniformly into the molten metal. Graphene dispersed Al composite foam describing compressive deformation under static loading [49], which reported ~28% improvement in specific energy absorption with the addition of 0.1 wt.% graphene nanoflakes. Al-foam manufactured by CYMAT (Canada and Fraunhofer IFAM processes) showed a significant rate of sensitive behavior, which was observed due to the microinertia effect [27]. In another study, Al-graphene composite foam was fabricated by a direct melt foaming technique [50]. The foaming agent and the graphene sheets were cryomilled before dispersing into the melt. The graphene sheets were detached in the melt and found along the side of the walls and stabilized the foam structure [31,32,33,34,50]. It is more beneficial to use fewer foam materials in escalated dynamic loads condition where energy absorption is high. Thus, there is a need to study systematically the deformation behavior of closed-cell Al-foam, under dynamic loading conditions to obtain optimized design of significant components.

In the confront research, the deformation of hybrid Al-SiC graphene composite foams under compressive loading conditions were observed, with a relative density (RD) range of 0.23 to 0.29. High strain rates utilizing SHPB apparatus were varied over the range of 500 s^−1^ to 2700 s^−1^. The strength and energy absorption phenomena of Al-foam materials are complex, and it is also difficult to understand the effect of different variables on its distortion conducts and energy absorption. It is essential to understand about influencing factors for the deformation behavior of hybrid composite foams especially inline to design for shock absorption. Analysis of Variance (ANOVA) optimizations are the statistical technique that helped the designer to find out the most significant parameters influencing any required response variable within a domain of input parameters. The effect of parameters such as mass, pore size, RD (relative density), and strain rates on the plateau stress and energy absorption were evaluated using ANOVA optimization process. The percent contribution of every parameter in estimating the properties were analyzed by using the ANOVA method. 

## 2. Materials and Experimental Methods

### 2.1. Production of Hybrid Aluminium Alloy Composite Foam Reinforced with SiC and Graphene 

**Figure 1:**
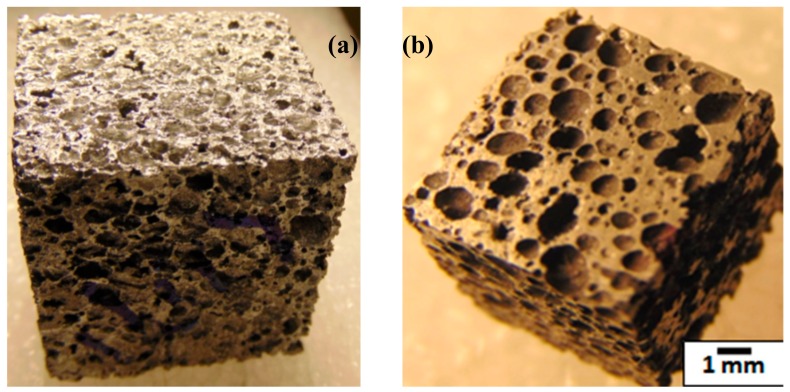
(**a**) Al foam block; (**b**) polished sample.

Closed-cell SiC (silicon carbide) and graphene reinforced hybrid Al alloy composite foams were produced through the solidification melt route process. In this manufacturing process, Al alloy (AA5083 which nominally contains 5.5% of Mg, 0.3% of Mn, 0.25% of Zn and rest is Al), was applied as matrix material. 15 wt.% of SiC particles (with size: 10–20 µm Make: Grindwell Norton, India) and 0.5 wt.% of graphene (supplied by the University of Missouri-Columbia) were blended in a molten Al alloy through casting. More graphene addition in Al melt would result in agglomeration of nano-graphene; hence, it was restricted to only 0.5 wt.%. SiC particles as a thickening agent were introduced to molten Al alloy, and nano-graphene was added to increase the strength of the cell walls of the foam. As a foaming agent, Calcium hydride was added (1.0 wt.%) in the Al alloy melt. After successful foaming, through compressed air, the metallic mold was cooled down. Figure 1a showed a block of Al hybrid composite foam and Figure 1b showed metallographically refined surfaces’ clearly depicted morphology of pores and their distribution.

### 2.2. Aluminium Foam Specimen Characteristics 

**Figure 2:**
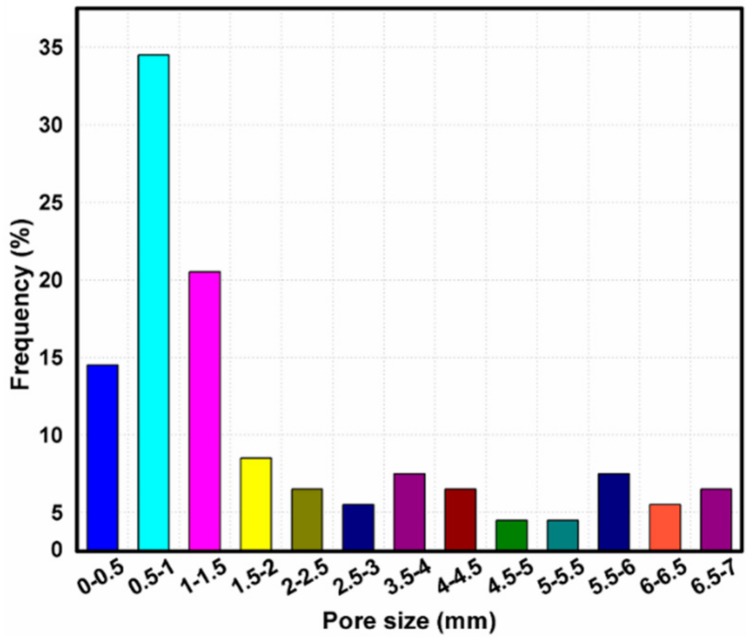
Pore size distribution of Al-SiC hybrid composite foam.

After assessment through the values of mass and volume of closed-cell Al-SiC hybrid composite foams, density was characterized. The RD of hybrid foams is the ratio of the foam material density to the aluminium alloy density (2.8 gm/cc). The average RD was determined in the range of 0.23–0.29 and the porosity in the range of 71%–77%. Microstructures of samples were prepared by employing a diamond cutting tool at lower speeds to avoid structural distortions. Before examining material samples under Scanning Electron Microscope (SEM), they were treated with conventional metallographical surface finish treatments including polishing, gold coating, etc. The shape of the pores was observed as equiaxed, and the size was nearly around 1 to 1.5 mm (Figure 2)—where the pore size was an approximate 2 mm (4% frequency) due to the collapsing behavior of pores. The size of more than 200 cells was measured randomly by using Image-Pro software, and the frequency (%) of pore size was plotted (Figure 2). It was noted from Figure 2 that 50% of the pores were in the size of around 0–1 mm, and 30% of them were of 1 to 2 mm and the rest of the pores were more than 2–7 mm. 

### 2.3. Split Hopkinson Pressure Bar Test

**Figure 3:**
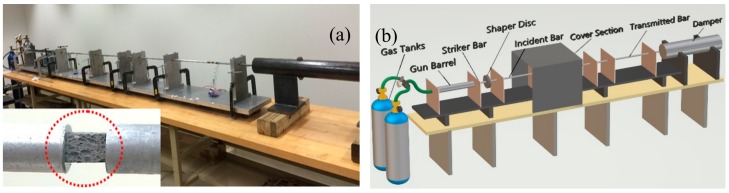
(**a**) SHPB (Split Hopkinson Pressure Bar) apparatus applied in the confront investigation as viewed from the transmitter bar end and foam specimen between the incident and transmitted bar shown in under doted circle; (**b**) major components of SHPB.

SHPB examines specimen’s behavior under compressive load at elevated strain values [51]. Figure 3a displays a SHPB model employed in this experimentation. The main components of the SHPB unit were a gas gun, incident bar, striker bar, transmission bar, and a damper shown in Figure 3b. The incident bar is built with 7075 Al alloy in T6 temper condition. The length of the incident bar was 182 mm, and the diameter was 12 mm. The transmitted bar was also built with 7075 Al alloy in T6 temper condition. The length of the transmitted bar was 137 mm, and the diameter was 12 mm. The striker velocity was varied in the range of 2–15 ms^−1^ depending on the strain rate. During impact, an elastic compression wave propagated in the direction of incident bar and the specimen. Incident bar and transmission bar grips the specimen to its opposite surfaces as shown in Figure 3a (marked with a dotted red circle). Once the wave reached the sample, the repetitive striking of waves plastically deforms the sample. The wave is partially propagated towards the transmission bar (transmitted pulse) and the remaining was mirrored towards the incident bar (reflected pulse), and the entire wave was received at strain gauges deployed on the respective bars. Stress–strain diagram was generated with the help of elastic strain values produced by the specimen. A pulse shaping technique was used to reduce stress wave dispersion and provide the stress equilibrium quickly. In the present investigation, a 145 tellurium-copper pulse shaper disk (with diameter 6.35 mm and thickness 1.59 mm) with five holes of 1 mm diameter, symmetrically drilled, was used as a pulse shaper, which is located in between the striker and incident bar. A detailed test procedure along with optimization of pulse shaper was reported elsewhere [52].

### 2.4. Optimization Methods

The present investigation optimization method (ANOVA) [37,53,54] was used to study the effect of parameters such as density, pore size, RD, strain rate, and mass on the plateau stress and energy absorption. In addition, it was used to study the most influential parameters which influenced basic output parameters such as plateau stress and energy absorption.

## 3. Results

### 3.1. Microstructural Studies

**Figure 4:**
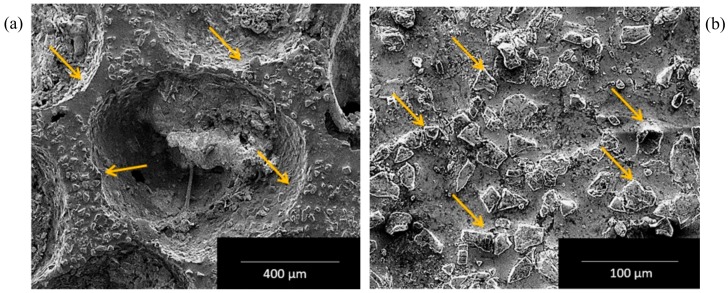
(**a**) SEM micrograph showing pores and SiC particles in the cell walls; (**b**) micrograph showing the distribution of SiC particles in the cell wall.

Figure 4a showed typical SEM microstructure of Al hybrid composite foam depicting pores and SiC particulates dispersion in the cell walls (arrow marked). The existence of SiC particulates in the cell walls provides stability and improves the strength ability of cell walls. A micrograph of the cell wall is depicted in Figure 4b, with the distribution of SiC particles (arrows marked). The SiC particles were equiaxed in shape and have sharp edges.

### 3.2. Strain Rate Deformation 

**Figure 5:**
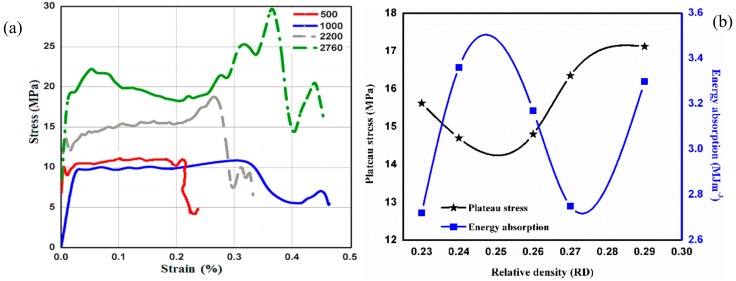
(**a**) compressive stress–strain diagram of Al hybrid composite foam with RD = 0.29 at different strain rate (500 s^-1^ to 2700 s^−1^); (**b**) stress and energy absorption relation with RD.

Investigating the behavior of Al-SiC hybrid composite foam under the dynamic compressive loading SHPB model was utilized where strain rates ranges from 500 s^−1^ to 2700 s^−1^. The RD of the Al-alloy hybrid composite foam specimens were expended from 0.23 to 0.29. The stress–strain graph evidently indicated elastic region before peak stress value and then, afterwards, the value of stress decreases until a constant value termed as plateau stress where the whole metal foam sample was deformed layer wise. The highest amount of stress a foam metal sample can bear is termed as yield stress. Figure 5a showed a stress–strain graph generated by Al-SiC hybrid composite foam specimen with RD 0.29. Moreover, the average value of plateau stress and energy absorption were considered from Table 1, and the plotted graph for stress and energy absorption relation with RD is shown in Figure 5b. Using ISO 13314 standard [37,55], the plateau stress was the average stress in the strain region of 10%–20% of the stress–strain curve. It clearly depicted that plateau stress was heightened with strain rates. For the strain rates ranging from 500 s^−1^ to 1000 s^−1^, the plateau stress was discovered closer to 10 MPa, which was raised to 20 MPa when strain rates were escalated to the range of 2300 s^−1^ to 2750 s^−1^. The stress–strain plot of an Al-SiC hybrid composite foam with RD of 0.23, 0.24, and 0.27 was demonstrated in a previous paper published by the authors [34,56]. In order to avoid repetition, only one diagram was shown and the values of plateau stress, yield stress, and energy absorption obtained for other strain rates were depicted in Table 1. 

The energy absorbed by Al-foam specimen under dynamic load conditions was determined by measuring the area below the stress–strain curve, up to a strain of 0.45 mm and their results were depicted in Table 1. The energy absorption, up to 0.45 strain, was considered to compare the relative effect of strain rate and density on the energy absorption. The tests were conducted at high strain rates and well below the densification strain to avoid any damage to the gun, incident, and transmitted bars and strain gauges. The strain was limited to the lock-up strain set during the test. In order to get an idea about the energy absorption by the foam samples, the densification was strained even in a dynamic condition. This was because of the fact where surging strain was primarily governed by the extent of porosity within the foam sample. 

### 3.3. ANOVA Analysis

The ANOVA was performed in MINITAB 2018, USA. The input responses were chosen as RD, mass, strain rates, pore size, and output of all factors in terms of energy absorption. The regression analysis of the current model was incurred for the response characteristics, specifically energy absorption. The response surfaces were plotted to study the influence of input process parameters i.e., RD, mass, strain rate, and pore size together with their second-order interactions on response characteristics. To study the significance of process variables towards energy absorption, ANOVA was performed. The input parameters for ANOVA analysis for energy absorption were taken from Table 1. It was also showed that the sequential model sum of squares test depicted how the terms of increasing complexity contribute to the model. The replicated design points, in the absence of a fit test, compare the residual error to pure error. Further tests summarize statistics given in this table, confirming that the quadratic model was finest as it exhibited low standard deviation, high “R-squared” values, and a low “PRESS” (Adequate precision). Table 2 indicated the results obtained from ANOVA, and it was noticed that variables such as RD, mass, strain rate, and pore size significantly affected both the mean value of energy absorption as well as a variation (S/N ratio; a signal to noise) in the energy absorption. The ANOVA technique indicated that the strain rate was the most significant input factor. Meanwhile, the most contributing factor for energy absorption was strain rates with 89.05%, and the least contributing factor was pore size with 0.13%. The second and third most contributing factors were RD (4.69%) and mass (3.94%).

**Figure 6:**
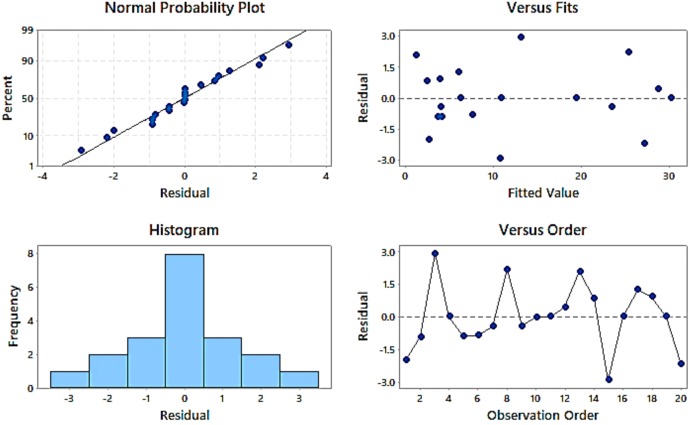
Residual plots obtained for energy absorption from the ANOVA test.

The pore size, mass, and RD were interdependent factors. In addition, the variation in pore size during this experiment was almost negligible. Therefore, the pore size contributed to the minimum of energy absorption. Figure 6 showed the normal probability plot of residuals which depicted that the observed values (energy absorption) lied reasonably close to the predicted line. It indicated that the relationship between the responses and the input parameters was appropriate, and the variation was within the limits. Most of the observed values (energy absorption) coincided with the predicted values. The histogram indicated that most of the observed values were of zero (0) residual with a frequency value of 8. The negative residual means the predictions were too high and positive residual indicated low prediction. It was seen from the present residual plots that the negative and positive residuals were not deviating much from the predicted line. The equal error variance was checked from the residual versus fit graph. Moreover, the fitted curve has been plotted by ANOVA results, whereas the residual was mostly in the range of ±1.0 and only in a few cases; it was observed more than ±1.0.

**Figure 7:**
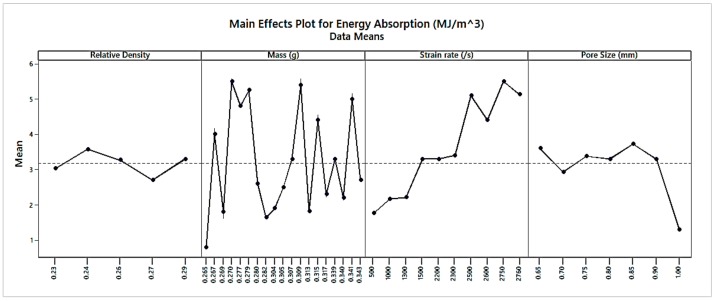
Main effect plot for energy absorption.

Figure 7 showed the main effects plot for energy absorption. The energy absorption first decreased then increased again, decreasing with an increase in RD. The main effects plot showed the effect of independent parameters contributed for energy absorption, excluding other parameters, while other parameters did not show evident variation as mass, RD and strain rate, which is more likely to contribute less. Non-uniform variation is observed concerning the mass. In most cases, energy absorption increased with an increase in strain rate. Energy absorption first decreased then increased, again decreasing with a gain in pore size. Analyzing plots, it was evident that energy absorption could be minimized using structures with the minimum strain rate; minimum relative density; minimum mass; and the maximum pore size. It was clear from Figure 7 that the energy absorption was lowest at 0.23 RD, 0.265 gm mass of the sample, 500 s^−1^ strain rate, and 0.65 mm pore size. 

**Figure 8:**
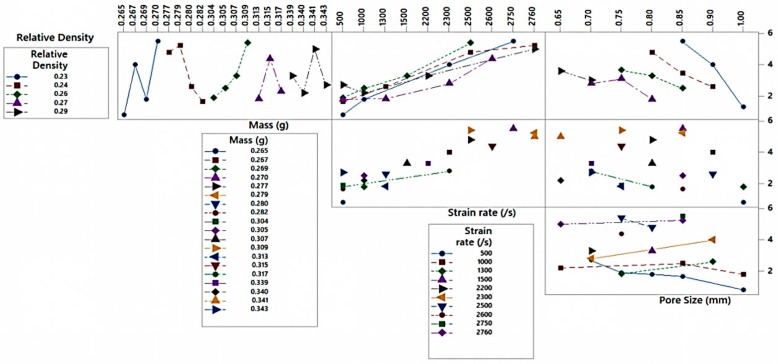
Interaction plot for energy absorption from the ANOVA test.

Figure 8 indicated a typical interaction plot obtained from ANOVA. The interaction plot depicted a convoluted relationship on various levels of dependent factors and independent factors on output, i.e., energy absorption of Al-foam. Independent variables such as RD, mass, strain rate, and pore size while working together produced different combined results by interacting with each other. An interaction plot displays correlation between independent and the dependent parameters i.e., energy absorbed at different levels. It also indicates influence of variables such as mass, strain rate, pore size, and RD on the output. The independent variables were not affected by each other, but they affect the dependent variable, which in this case was the energy absorption. It was also to be mentioned here that two independent variables combined with each other (i.e., strain rate and pore size) could affect the dependent variables (energy absorption). 

## 4. Discussion

A lot of research has been conducted to study the influence of strain rates on the deformation response of Al-foam under dynamic loading, and it was reported that the deformation response of Al-foam was almost insensitive to the strain rates [26,55,57,58,59]. The ability of Al-foam to absorb energy depends on the area below the stress–strain curve until the strain rate starts surging. Thus, in the case of aluminium foam, one should be aimed for enhancing the plateau stress by keeping the densification strain rate higher, to attain a high degree of energy absorption. This could be accomplished by modifying the cell wall characteristics by varying the cell geometry and by introducing alloying elements or high strength phases. Furthermore, optimizing the cell size leads to increasing the surface area, improving plateau stress and the ability to absorb energy. Lately, a study that introduced dispersion of carbon nanotubes [60] and graphene nano-sheets in liquid metal strengthened the cell walls and led to enhancement of the plateau stress and energy absorption of Al-foam. Hence, it is required to understand their deformation behavior under compression at different strain rates for metal foam applications to absorb impact. The deformation behavior of Al-foam under compression at quasi-static conditions was examined and now substantially understood. However, its behavior at a higher strain rates using the SHPB unit was examined and explained the deformation mechanism by some researchers [47,48], but still there was a lack of uniform understanding. While manufacturing Al alloy foam, elevated temperatures dissociated metal hydride and released hydrogen that gives molten metal its foam structure. Subsequently, the structure needed to stabilize and cool down immediately afterwards; otherwise, the complete structure can be drawn off and collapse instantly. Usually, to improve viscosity and facilitate sturdiness to the Al-foam structure, Ca (calcium) or ceramic particles (SiC, Al_2_O_3_, etc.) were dispersed in the liquid metal. In the confront experiment, viscosity was improved with ensuring the stability of the Al-foam structure, when 15 wt.% SiC particulates were reinforced to the molten Al alloy. Figure 4 showed SEM micrographs of the foam structure and SiC particulates dispersed in the wall structure, which stabilized the foam structure and hindered the draining of liquid metal. Throughout the deformation under the influence of compression, foam structure encountered several phenomena such as compression at successive layers, cell wall bending, shearing, crushing, and compaction. The yield stress occurred at the point when deformation just started. The topmost layer of the specimen was subjected to yield stress, at the very beginning of the deformation. The stress was then transferred to the next layer, after the top layer was compressed. Moreover, keeping the stress value constant, the deformation of the successive layer takes place, while, following this, the entire foamed specimen (~80% of the sample up to densification strain) got compressed by escalating strain value—as a consequence of which the inflated cells began to densify by getting in contact with the adjoining cell wall without varying plateau stress value. The specimen material encountered structural distortion at the wall in the direction perpendicular to the load where the cell started collapsing plastically. Resulting in the lowering of plateau stress as depicted, the presence of SiC particulates in the cell walls greatly affected the elastic properties and initiated crack formation in the matrix or particle interface in Figure 5.

In the current scenario, the compressive deformation behavior of Al-SiC hybrid composite foam was examined in the strain rates of from 500 s^−1^ to 2760 s^−1^. For better understanding, the entire range of strain rates was separated in three sections, (i) 500 s^−1^ –1500 s^−1^, (ii) 1500 s^−1^ –2000 s^−1^, and (iii) 2000 s^−1^ –2760 s^−1^. It was discovered that, up to a strain rate of 1500 s^−1^ with RD ranging from 0.23 to 0.29, the plateau stress encountered was 10–13 MPa. Further shooting up the strain rate to 2000 s^−1^, the plateau stress was found to be 14–18 MPa and the ultimate strain rate at 2760 s^−1^; the plateau stress was increased to 20–22 MPa. Corresponding results were proposed by Kang Ying-an et al. [61], where Al-SiC composite foam carrying material density of 0.457 gm/cc possesses plateau stress at 3.4 MPa while maintaining strain rates at 1600 s^−1^ and yield stress at 2.8 MPa. The higher plateau stress and energy absorption of the Al-form obtained in the present investigation might be due to the presence of graphene in the cell walls. It was recently claimed by Yadav et al. [62] that the addition of SiC and CNT (Carbon naotube) together in Al alloy matrix exhibited significantly higher strength than the one where SiC and CNT are added separately. It is reported that SiC acted as a secondary reinforcing agent, which caused the better distribution of CNTs and the CNTs entangled SiCs in the matrix causing stronger interface bonding. When SiC and CNT were added separately, the distribution of CNT was poor and the interface bonding with matrix was relatively poor. Similarly, it was expected (as graphene and CNT both have almost similar chemical and physical characteristics) that the addition of graphene and SiC together improved the distribution of graphene and SiC particles and the interfacial bond between the matrix material and graphene. Similar kinds of observation were depicted in Al-SiC graphene composite made through ultrasonic stirring [63]. It was also reported that the presence of graphene nanoflakes in Al-foam reduced the pore size, improved pore distribution and refined pore morphology, which, in turn, enhanced the properties [49]. The strain rate strengthening phenomena was clearly discernible in the current experimentation. The results generated for hybrid Al-alloy composite foam, on account of energy absorbed, RD, and strain rates, showed that, up to a strain rate of 1500 s^−1^, the energy absorption of the foam material encountered was 1.8–2.6 MJm^−3^, and, at strain rates of 1500 s^−1^–2000 s^−1^, the energy absorption was discovered in the range of 3.0 to 4.0 MJm^−3^. Furthermore, enhancing the strain rate to 2000 s^−1^–2760 s^−1^, the energy absorbed was ranged from 5.0 to 5.4 MJm^−3^. These results were confirmed by the predicted values obtained by ANOVA analysis. The current investigation depicted that plateau stress and energy absorbed by Al-alloy hybrid composite foam was highly influenced by strain rates during dynamic loading. It has been proposed that the pore morphology, cell wall strengthening, and Al-SiC graphene inclusions in the cell wall might be the reasons for strengthening. Dannemann and Lankford et al. [59] compared increase in strength with the movement of gas through fractured cell walls, whilst Elnasri et al. [64] called out concern regarding shock waves for improved material strength results. Investigating the whole gamut of strengthening, the parameters accountable for the flow stress of the material could be envisaged as strain rates and the cell structural characteristics i.e., the wall material, morphology, orientation, etc. All the parameters acted at once, enhancing the plateau stress and making the material sustainable in order to absorb additional energy while getting plastically deformed. Graphene nanosheets were deployed with the SiC particulates to enhance material strength as a consequence of a much stronger bond developed at the interface of Al-SiC and graphene [59]. It was reported [49] that the inclusion of 0.3% of nano-sheets of graphene in Al-alloy improved the tensile strength by 62% compared to the non-reinforced alloy. The present results were well validated through the predicted results obtained by ANOVA analysis. The strain rate contribution, in enhancing the energy absorption, was found to be the highest (89%) and nominal contribution of RD observed on the order of 4.69% (Table 2). This type of analysis was found to an excellent methodology for designing an Al- SiC graphene hybrid composite foam for a specific application using sets of data at randomly selected input variables. 

## 5. Conclusions

The Al-SiC graphene hybrid composite foam with pore size ranging from 0.5 to 2 mm and RD of 0.23 to 0.29 was examined under dynamic loading at different strain rates in a range of 500 s^−1^–2760 s^−1^ through utilizing a SHPB Unit. The following parameters were deliberated from this work:

▪ The compression tests of foam samples indicated the plateau stress of 10 MPa at a strain rate of 500 s^−1^ and 20 MPa at a strain rate of 2760 s^−1^ and energy absorption was found in the range of 1–5 MJm-3.
▪ The plateau stress and energy absorption were sensitive to strain rate and insensitive to RD used in the presented work.
▪ There was a two-fold gain in plateau stress and a five-fold gain in energy absorption with an increase of strain rate from 500 s^−1^ to 2750 s^−1^
▪ ANOVA was utilized for evaluating the most prominent parameter to contribute to energy absorption. In addition, it has been seen that the contributions of the strain rate on energy absorption were 89.05%, which was the highest amongst the parameters.
▪ RD had less influence on controlling the energy absorption, and it was observed to be only 4.69%. Predictions from ANOVA analysis well agreed with the experimental data.

The current report has brought to the closure by understanding the fact that there is plenteous scope existed to explore enhancement in the material properties of closed-cell Al foam with the inclusion of nano-sheets of graphene. The presence of 0.5 wt.% graphene in Al-15wt.% SiC composite enhanced the strength of the foam structure considerably and this opened a new avenue for further study in this direction.

## Figures and Tables

**Table 1:** Relative density, plateau stress, yield stress, energy absorption of Al composite hybrid foam with graphene at various strain rates and input data for ANOVA.

RD	Pore Size (mm)	Strain Rate (s^−1^)	Mass (gm)	Yield Stress (MPa)	Plateau Stress (MPa)	Energy Absorption (MJm^−3^)
0.23	1	500	0.265 ± 0.13	8 ± 0.10	8.5 ± 0.11	0.8 ± 0.09
	1	1000	0.269 ± 0.12	10 ± 0.11	12 ± 0.13	1.8 ± 0.19
	0.90	2300	0.267 ± 0.14	18 ± 0.21	20 ± 0.24	4.0 ± 0.20
	0.85	2750	0.270 ± 0.12	22 ± 0.23	22 ± 0.25	4.3 ± 0.23
0.24	0.85	500	0.282 ± 0.13	10 ± 0.11	9.1 ± 0.12	1.65 ± 0.12
	0.90	1300	0.280 ± 0.10	14 ± 0.16	13.2 ± 0.15	2.6 ± 0.13
	0.80	2500	0.277 ± 0.15	18 ± 0.20	21.5 ± 0.21	4.3 ± 0.22
	0.85	2760	0.279 ± 0.15	23 ± 0.25	25 ± 0.29	4.9 ± 0.26
0.26	0.75	500	0.304 ± 0.14	11 ± 0.13	9.1 ± 0.11	1.90 ± 0.14
	0.85	1000	0.305 ± 0.13	13 ± 0.15	12.5 ± 0.14	2.1 ± 0.22
	0.80	1500	0.307 ± 0.16	15 ± 0.17	15 ± 0.16	3.3 ± -0.28
	0.75	2500	0.309 ± 0.14	20 ± 0.22	22.6 ± 0.24	5.4 ± 0.29
0.27	0.80	500	0.317 ± 0.13	12 ± 0.13	9.5 ± 0.11	1.8 ± 0.16
	0.75	1300	0.313 ± 0.15	14 ± 0.16	13.8 ± 0.14	1.82 ± 0.17
	0.70	2300	0.317 ± 0.16	15 ± 0.19	22.1 ± 0.21	2.80 ± 0.21
	0.75	2600	0.315 ± 0.12	18 ± 0.20	20 ± 0.20	4.40 ± 0.25
0.29	0.70	500	0.343 ± 0.13	10 ± 0.11	11 ± 0.13	2.7 ± 0.22
	0.65	1000	0.340 ± 0.16	11 ± 0.13	13.5 ± 0.15	2.2 ± 0.24
	0.70	2200	0.339 ± 0.13	14 ± 0.16	19 ± 0.16	3.3 ± 0.26
	0.65	2760	0.341 ± 0.15	22 ± 0.23	25 ± 0.29	5.0 ± 0.28

**Table 2:** Results obtained from ANOVA analysis.

Source	DF	Seq SS	Contribution	Adj SS	Adj MS	F-Value	*p*-Value
RD	4	89.13	4.69%	42.36	10.589	1.02	0.493
Mass (gm)	1	74.73	3.94%	5.54	5.536	0.53	0.506
Strain rate (s^−1^)	9	1690.80	89.05%	1035.42	115.047	11.06	0.017
Pore size (mm)	1	2.52	0.13%	2.52	2.518	0.24	0.649
Error	4	41.62	2.19%	41.62	10.406		
Total	19	1898.79	100.00%				

Notes: DF: Degree of freedom; Seq SS: Sequential sum of square; Adj SS: Adjusted sum of square; Adj MS: Adjusted mean of square; F: Statistical test; P: Probability test
